# Adverse effects of systemic therapy in a patient with urothelial bladder cancer and chronic kidney disease – a case report

**DOI:** 10.3389/fonc.2026.1733345

**Published:** 2026-07-02

**Authors:** Martyna Najmrocka, Aleksandra Semeniuk, Rafał Stec

**Affiliations:** Klinika Onkologii, Warszawski Uniwersytet Medyczny Wydzial Lekarski, Warsaw, Poland

**Keywords:** chronic kidney disease, glucocorticoid toxicity, immune-related adverse events, nivolumab, urothelial carcinoma

## Abstract

For patients with urothelial bladder cancer who are ineligible for cisplatin-based chemotherapy, particularly those with chronic kidney disease (CKD), immune checkpoint inhibitors (ICIs) have become an important therapeutic alternative. However, these agents may cause immune-related adverse events (irAEs) that can mimic CKD progression and complicate clinical management. We report the case of a 72-year-old man with type 2 diabetes mellitus and CKD who underwent cystoprostatectomy for locally advanced urothelial carcinoma. Following confirmation of PD-L1 positivity, adjuvant nivolumab was initiated at a dose of 240 mg every two weeks. After three treatment cycles, the patient developed diffuse myalgia, progressive weakness of all limbs, and paresthesia of the upper extremities. Laboratory results showed leukocytosis with neutrophilia and elevated alanine aminotransferase (100 U/L), C-reactive protein (119 mg/L), and troponin (113 ng/L), with normal creatine kinase activity. Electrocardiography and echocardiography excluded ischemia and myocarditis, while electromyography was unremarkable. High-dose intravenous glucocorticosteroids (1 mg/kg) led to clinical improvement, but symptom recurrence during tapering required re-escalation of immunosuppressive therapy. Multidisciplinary evaluation revealed chronic steroid toxicity and secondary testosterone deficiency. The patient continued on a gradual steroid taper combined with testosterone replacement and rehabilitation, achieving steady functional recovery under specialist follow-up. The presentation was consistent with ICI-induced muscular and endocrine toxicity, with no evidence of myocarditis or neuromuscular transmission disorder. This case underscores the diagnostic complexity of distinguishing irAEs from CKD progression and other comorbidities, highlighting the importance of coordinated input from oncology, nephrology, endocrinology, neurology, and cardiology teams. Adjuvant nivolumab remains a valuable therapeutic option for cisplatin-ineligible urothelial carcinoma patients with CKD, but its safe use requires vigilant monitoring, timely immunosuppression, and cautious steroid tapering. This report contributes to the limited body of evidence on ICI safety in patients with renal impairment and provides practical insights for multidisciplinary management in this challenging clinical context.

## Introduction

Bladder cancer is the 10th most common malignancy worldwide, with urothelial carcinoma accounting for approximately 90% of cases (1). In 2020, 573,278 new cases of bladder cancer were reported, and projections estimate that by 2040, the number will rise to 913,002. The disease occurs 3–4 times more frequently in men than in women and mainly affects older individuals (2). In Europe, around 200,000 new cases are diagnosed annually, while in Poland, the incidence is approximately 8,000–9,000 cases per year, making bladder cancer a significant public health concern.

The standard treatment for advanced urothelial carcinoma remains cisplatin-based chemotherapy. However, many patients, particularly those with chronic kidney disease (CKD), are ineligible for such therapy. In recent years, immune checkpoint inhibitors (ICIs) have been introduced—anti-PD-1 antibodies (nivolumab, pembrolizumab) and anti-PD-L1 antibodies (atezolizumab, avelumab) to bladder cancer treatment (3). These drugs have markedly improved outcomes in patients who previously had limited therapeutic options, as it does not require preserved renal function (5). Their efficacy reaches 20–30% objective response rates, and in some patients leads to durable remission. At the same time, their use carries the risk of immune-related adverse events (irAEs) affecting multiple organ systems (4).

In addition, its use poses specific diagnostic challenges as renal function deterioration may result either from the natural progression of CKD or from immunotherapy-related adverse events (6). Furthermore, the use of glucocorticosteroids in the management of irAEs may worsen metabolic control and impose additional burden on the patient.

The literature provides many reports of immune-related adverse events in patients with melanoma or lung cancer, while data concerning urothelial carcinoma remain scarce. Reports on patients with concomitant CKD are even rarer, despite the fact that for many of them, immunotherapy is the only feasible alternative to cisplatin-based chemotherapy. There is a lack of publications highlighting the challenges in distinguishing between irAEs and CKD progression, as well as those discussing practical management strategies in this patient population (7).

The present case describes a patient with urothelial carcinoma and chronic kidney disease who developed severe adverse events during nivolumab therapy, illustrating the diagnostic and therapeutic challenges in this group of patients.

## Clinical history

A 72-year-old male suffering from diabetes mellitus and chronic kidney disease (CKD Stage 3, diagnosed based on KDIGO criteria with a baseline estimated glomerular filtration rate (eGFR) of 40 mL/min/1.73m² maintained for over 3 months) was diagnosed with locally advanced urothelial carcinoma of the bladder. According to the preoperative computed tomography (CT) report, the patient presented with an advanced, muscle-invasive tumor in stage cT3NXM0. Notably, no signs of hydronephrosis were reported, indicating that his pre-existing CKD was secondary to diabetic nephropathy rather than post-renal obstruction. He was not qualified to cisplatin-based neoadjuvant chemotherapy by multidisciplinary team and cystoprostatectomy was the main therapeutic option proposed to him. Following cystoprostatectomy, positive PD-L1 expression was confirmed so the patient was qualified for adjuvant therapy with nivolumab at 240mg every 2 weeks. At the start of therapy, the patient was in good performance status, ECOG 1.

Timeline of Clinical Events:

- Month 0: Diagnosis of locally advanced muscle-invasive urothelial carcinoma; deemed ineligible for cisplatin-based chemotherapy due to baseline CKD.
- Month 1: Radical cystoprostatectomy; histopathology confirms tumor stage and positive PD-L1 expression.
- Month 2: Initiation of adjuvant immunotherapy with nivolumab (240 mg every 2 weeks).
- Month 3.5 (After 3 cycles): Onset of severe neuromuscular symptoms, limb weakness in grade 3 according to The NCI Common Terminology Criteria for Adverse Events 5.0 (CTC AE), elevated troponin and ALT in grade 1 CTC AE; hospitalization and initiation of IV glucocorticosteroids (1mg/kg) with clinical improvement.
- Month 4–5: Symptom recurrence during steroid tapering; re-escalation of immunosuppression; diagnosis of chronic corticosteroid toxicity and testosterone deficiency.
- Month 6+: Initiation of testosterone replacement therapy and rehabilitation; gradual clinical improvement during outpatient follow-up.

### Treatment

Adjuvant immunotherapy with nivolumab was initiated according to current guidelines. Initially, the patient tolerated the treatment well, and patient denied any side effects. Adjuvant immunotherapy with nivolumab was initiated according to current local guidelines and the CheckMate 274 trial protocol, utilizing the approved scheduling of 240 mg intravenously every 2 weeks. This standard dose was selected instead of a 3-weekly regimen, which is not indicated for single-agent adjuvant nivolumab in muscle-invasive urothelial carcinoma.

### Adverse events

After 3 cycles of treatment, the patient developed joint and muscle pain along with weakness of the upper and lower limbs. He had no problems with breathing, unfortunately he was unable to walk on his own. In addition, he reported numbness and tingling, mainly in the upper limbs and he was unable to raise his arms. Physical examination revealed decreased muscle mass and decreased muscle strength. The patient was not taking any medications known to cause myositis. Laboratory results showed leukocytosis with neutrophilia, elevated ALT (100 U/L), elevated troponin (113 ng/l), and CRP (119 mg/l), with normal CK levels. The kidney function remained stable. ECG and echocardiography revealed no ischemic changes and no signs related to myocarditis, and EMG/SFEMG studies were unremarkable. Intravenous glucocorticosteroids were administered at 1mg/kg, leading to clinical improvement. After achieving a satisfactory improvement, the dose of glucocorticosteroids was gradually reduced according to local guidelines. However, after tapering the steroid dose, symptoms recurred mainly with a predominant general weakness, necessitating re-escalation of immunosuppression.

### Diagnostic work-up

The patient underwent multidisciplinary evaluation, including neurological, cardiological, pulmonological and endocrine consultations. Further diagnostics revealed chronic glucocorticosteroid toxicity and testosterone deficiency.

### Outcome and follow-up

The patient continued on tapered doses of glucocorticosteroids with the addition of testosterone replacement therapy. He was referred for rehabilitation and remains under follow-up, with clinical improvement observed under specialist outpatient care. Unfortunately, the patient’s performance status remained impaired and he moved around using a specialized walker.

## Discussion

In this patient with urothelial carcinoma and chronic kidney disease, nivolumab therapy led to muscle and endocrine-related adverse events. These complications required treatment interruption, administration of glucocorticosteroids, and further specialized evaluation.

Immune checkpoint inhibitor–related adverse events can affect multiple organs. The most frequently reported include dermatologic toxicity, colitis, pneumonitis, and endocrinopathies (8). Muscular complications such as myopathy and myositis are less common but may be severe and require aggressive management. Cases of nivolumab-associated myasthenia gravis and myopathy have been reported (9). Renal toxicity occurs in approximately 2–5% of patients, most commonly presenting as interstitial nephritis (10).

The majority of reports concern patients with melanoma or lung cancer, whereas descriptions of adverse events in urothelial carcinoma remain limited. Reports involving patients with concomitant CKD are exceptionally rare, despite this being a particularly vulnerable group in whom renal function decline may reflect either CKD progression or irAEs (7).

An important lesson from this case is the need to prioritize careful clinical assessment and physical examination when evaluating suspected immune-related adverse events. Despite the severity of the patient’s symptoms, the results of laboratory and additional diagnostic investigations did not consistently correlate with the clinical presentation. Creatine kinase levels remained within the normal range, electrophysiological studies were unremarkable, and no definitive evidence of myocarditis or other organ-specific immune-mediated injury was identified. Therefore, therapeutic decisions were driven primarily by the patient’s clinical condition rather than by objective laboratory abnormalities. This observation highlights that immune-related toxicities may present with nonspecific findings and that the absence of characteristic laboratory abnormalities does not exclude clinically significant toxicity.

Furthermore, adverse effects of concomitant medications should always be considered in the differential diagnosis of new or recurrent symptoms. In the present case, functional deterioration, fatigue, muscle weakness reported during GKS tapering may be caused by recurrence of myopathy or by GKS toxicity during prolonged therapy. Consequently, it was not possible to unequivocally determine whether the recurrence of symptoms during follow-up reflected reactivation of an immune-mediated process or complications related to chronic corticosteroid exposure. This diagnostic uncertainty emphasizes the complexity of managing immune checkpoint inhibitor–associated toxicities and underlines the importance of continuous multidisciplinary evaluation throughout treatment and follow-up.

This case highlights the necessity of individualized management in patients with urothelial carcinoma and comorbid CKD. Optimal care requires close multidisciplinary collaboration, including endocrine, cardiological, and neurological input. Early recognition of adverse events and timely initiation of immunosuppressive therapy are essential.

From a pharmacological perspective, the use of nivolumab in patients with renal impairment is justified by its metabolic pathway. Unlike conventional cisplatin-based chemotherapy, nivolumab is not excreted or metabolized by hepatic cytochrome P450 enzymes. Instead, it is eliminated through nonspecific receptor-mediated endocytosis and subsequent lysosomal proteolytic degradation into small peptides and amino acids. Consequently, pre-existing CKD does not significantly alter the pharmacokinetics or systemic clearance of nivolumab, making it a theoretically safe therapeutic alternative in this vulnerable population. Evaluating laboratory anomalies in this patient required careful consideration of the systemic effects of renal failure. Chronic kidney disease is inherently associated with immune dysregulation, characterized by a functional impairment of the white blood cell system. Although patients with CKD often exhibit chronic low-grade inflammation, their leukocytes demonstrate impaired chemotaxis, phagocytosis, and adherence, which compromises their primary immune response. In our patient, the observed leukocytosis with neutrophilia presented a diagnostic challenge. It likely reflected a combination of this baseline uremic immune dysregulation and the compounding effect of corticosteroid-induced neutrophil demargination, rather than an active underlying infection.

The current data from the literature regarding the clinical effects of ICIs on patients with pre-existing CKD are summarized in **Table 1**.

**Table 1:** Summary of literature data on the clinical effects of immune checkpoint inhibitors (ICIs) in patients with pre-existing chronic kidney disease (CKD).

Study/author (year)	Patient population & tumor type	Pre-existing CKD status	Key findings & clinical effect on ICI recipients
Seethapathy et al. (2019) (6)	Large cohort (n=1,016) with various malignancies (lung, melanoma, urothelial cancer).	Baseline eGFR < 60 mL/min/1.73m²	Patients with a lower baseline eGFR had a significantly higher risk of developing acute kidney injury (AKI) during ICI therapy, often complicating the distinction between CKD progression and immune-related nephritis.
Kanzaki et al. (2020)	Retrospective study on advanced solid tumors treated with anti-PD-1/PD-L1 agents.	CKD Stages 3 to 5 (severe renal impairment)	Demonstrated that pre-existing severe CKD did not significantly increase the overall incidence or severity of general immune-related adverse events (irAEs) or specific renal toxicity compared to patients with normal kidney function.
Torniai et al. (2025) (7)	Case report of a patient with sarcomatoid urothelial carcinoma receiving pembrolizumab.	Concomitant severe renal impairment	Highlighted the clinical complexity of managing overlapping, severe systemic irAEs (dermatomyositis) while closely balancing immunosuppressive treatments and maintaining renal function monitoring.
Present Case (2026)	72-year-old male with muscle-invasive urothelial bladder carcinoma receiving adjuvant nivolumab.	CKD (Diabetic nephropathy), baseline eGFR decreased	Adjuvant nivolumab triggered severe muscular (myopathy-like) and endocrine toxicities. The mandatory high-dose systemic glucocorticoid therapy severely disrupted metabolic and glycemic control, imposing an indirect but significant clinical burden on the patient’s pre-existing diabetic CKD status.Adjuvant nivolumab triggered severe muscular and endocrine toxicities. Because the patient’s physiological reserves were already severely compromised by pre-existing CKD, the mandatory high-dose systemic glucocorticoid therapy carried an exceptionally high risk, drastically disrupting metabolic and glycemic control and imposing a severe secondary clinical burden on the patient.

### Strengths and novelties

The primary novelty of this case report lies in describing the clinical course of adjuvant nivolumab therapy in a patient with muscle-invasive urothelial carcinoma and pre-existing chronic kidney disease (CKD). A population systematically excluded from pivotal clinical trials. While most literature focuses on metastatic settings or other malignancies, this report provides rare, real-world evidence on the safety profile and the specific diagnostic dilemma of distinguishing between CKD progression and multi-organ immune-related adverse events (irAEs). Furthermore, it highlights the metabolic challenges of using high-dose glucocorticosteroids to treat irAEs in a patient with concomitant diabetes and CKD.

### Limitations

This study has certain limitations. As a single-patient case report, the findings cannot be widely generalized. Due to the retrospective nature of this report and the fact that the initial diagnostics and surgery were performed at an external institution, the original digital imaging files (CT/MRI) and microscopic figures for PD-L1 staining were unavailable for reproduction. However, all diagnostic data and cut-off values used for treatment qualification were verified based on official, certified medical and histopathological documentation. Lastly, a renal biopsy was not performed, which limited our ability to definitively rule out subclinical immune-related interstitial nephritis versus diabetic nephropathy progression.

## Conclusion

Immunotherapy represents an effective treatment option for patients with urothelial carcinoma ineligible for cisplatin-based chemotherapy. However, in patients with concomitant chronic kidney disease, its use poses specific diagnostic and therapeutic challenges. This case adds to the limited body of literature and underscores the need for further research on the safety and management of immunotherapy in this population.

## Data Availability

The original contributions presented in the study are included in the article/supplementary material. Further inquiries can be directed to the corresponding author.
